# Septo-optic Dysplasia Plus Syndrome

**DOI:** 10.7759/cureus.3727

**Published:** 2018-12-13

**Authors:** Alejandro Gutierrez-Castillo, Amado Jimenez-Ruiz, Melissa Chavez-Castillo, José Luis Ruiz-Sandoval

**Affiliations:** 1 Miscellaneous, Instituto Tecnológico Y De Estudios Superiores De Monterrey, Monterrey, MEX; 2 Neurology, Instituto Nacional de Ciencias Medicas y Nutricion Salvador Zubiran, Ciudad de Mexico, MEX; 3 Pediatric Neurology, Instituto Nacional De Pediatría, Mexico City, MEX; 4 Neurology, Hospital Civil De Guadalajara "Fray Antonio Alcalde", Guadalajara, MEX

**Keywords:** septo-optic dysplasia, seizure, optic nerve hypoplasia, schizencephaly, epilepsy, growth hormone deficiency, cortical dysplasia, septum pellucidum agenesis, polymicrogyria, hypoplasia of corpus callosum

## Abstract

Septo-optic dysplasia plus is a rare congenital syndrome characterized by the classic triad of optic nerve hypoplasia, hypothalamic-hypophyseal dysfunction, and midline abnormalities, with associated malformations of cortical development. Clinical manifestations include optic nerve disease, epilepsy, intellectual delay, and endocrine dysfunction. We present the case of an 18-year-old man with a history of seizures, growth hormone deficiency, and optic nerve disease that was diagnosed with septo-optic dysplasia plus syndrome with characteristic imaging findings.

## Introduction

Septo-optic dysplasia (SOD), also known as “de Morsier’s Syndrome,” was first reported in 1956 by the Swiss neurologist George de Morsier who described the following triad: hypoplasia/dysplasia of the optic nerve, hypothalamic-hypophyseal dysfunction, and midline abnormalities (dysgenesis of corpus callosum and/or septum pellucidum) [1]. Years later, the term SOD-plus was suggested to differentiate SOD with associated malformations of cortical development [2].

Despite the current controversy regarding the definite etiology of this syndrome, the most accepted hypothesis is the vascular disruption sequence, especially [3-4], an anomaly in the proximal trunk of the anterior cerebral artery [4]. Although most of the cases are sporadic, less than 1% of cases have been associated with mutations in the following genes: HESX1, SOX2, SOX3, or OXT2 [5-6].

In Mexico, there is no information available on its epidemiology, however, previous studies in other countries have shown a prevalence of 1.9-5.4 per 100,000 live births and an increasing incidence of 113.3 per 100,000 live births [7]. There is no gender predilection, but a young maternal age seems to be a common risk factor [7-8].

We present the case of a young adult with SOD-plus with associated ophthalmic, neurologic, and endocrine manifestations.

## Case presentation

An 18-year-old male patient was referred to our unit for the management and follow-up of epilepsy diagnosed during childhood. The patient had focal seizures evolving to bilateral tonic-clonic seizures treated initially with valproic acid. Seizures were well-controlled until a year before presentation when the seizure pattern recurred.

His family history was incomplete due to the absence of the father during upbringing. The patient’s mother was 17 years old when she became pregnant. The medical history was relevant for short stature due to growth hormone (GH) deficiency, with no substitution during childhood due to financial limitations.

The general physical examination revealed short stature (133 centimeters) and low weight (45 kilograms) with a body mass index (BMI) of 20.93 (normal BMI: 18.5-24.99). His vital signs were within normal limits. The neurological examination was relevant for complete right eye blindness with atrophy of the right optic nerve and diminished visual acuity in the left eye; a right horizontal nystagmus was found in the neutral position, which worsened with right lateral gaze. Muscle strength was diminished in the left hemibody with ipsilateral hyperreflexia and extensor plantar response.

**Figure 1:**
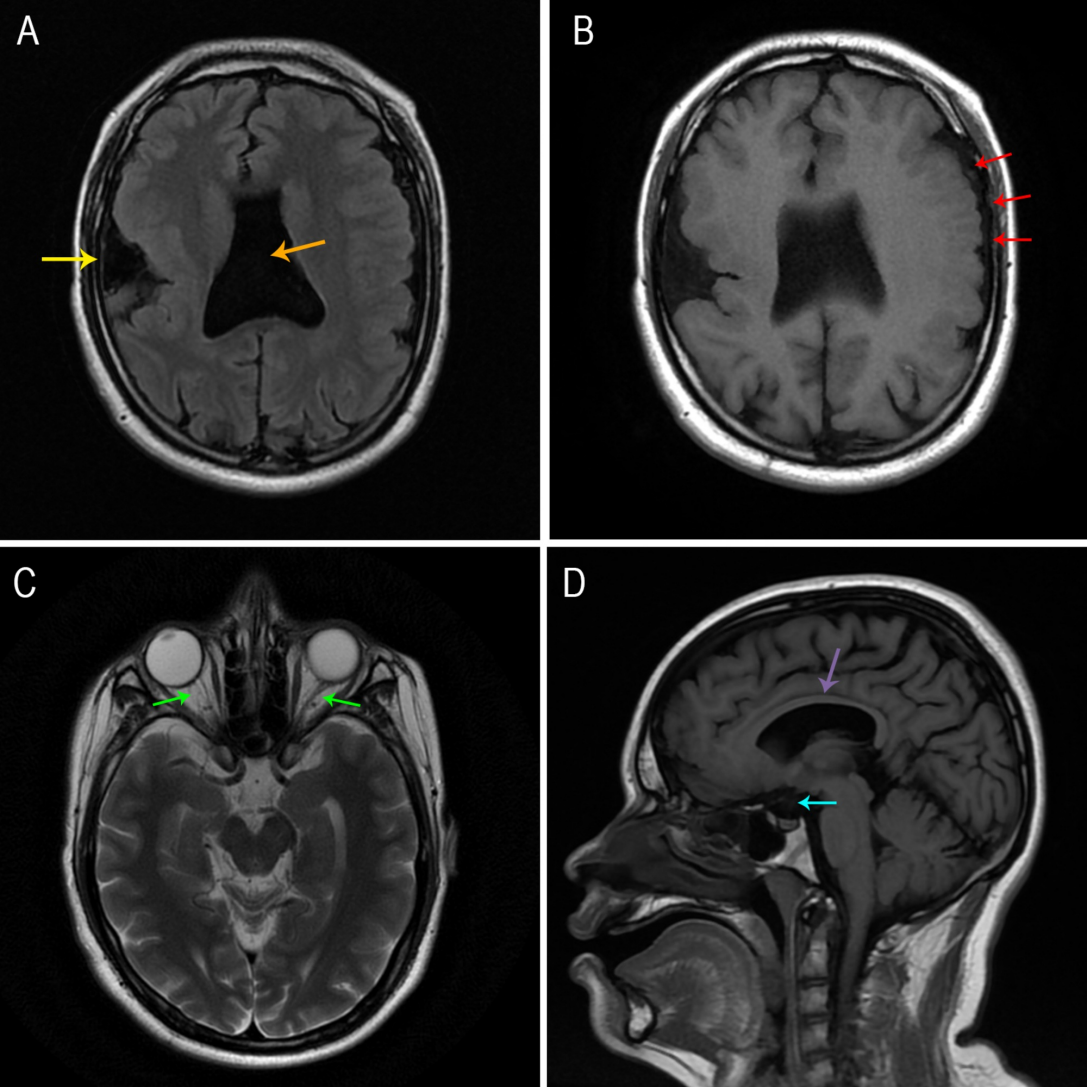
Magnetic resonance imaging Panel A: T2-weighted fluid-attenuated inversion recovery (FLAIR) sequence, axial view. Agenesis of the septum pellucidum, which conditions a “mono-ventricle” appearance (orange arrow) and closed-lip schizencephaly in the right frontal-temporal area, associated with generalized cortical dysplasia of the surrounding parenchyma (yellow arrow). Panel B: T1-weighted FLAIR sequence, axial view. Polymicrogyric appearance and disposition in the left transitional frontal-parietal area (red arrows). Panel C: T2-weighted periodically rotated overlapping parallel lines with enhanced reconstruction (PROPELLER) sequence, axial view. Hypoplasia of both optic nerves (green arrows). Panel D: T1-weighted FLAIR sequence, sagittal view. Hypoplasia of the corpus callosum (purple arrow) and hypoplasia of the pituitary stalk (blue arrow).

Magnetic resonance imaging (MRI) reported the following findings:

- Agenesis of the septum pellucidum, which conditioned a “mono-ventricle” appearance and agenesis of the anterior portion of the falx cerebri

- Closed-lip schizencephaly in the right frontal-temporal area, associated with generalized cortical dysplasia of the surrounding parenchyma

- Hypoplasia of the corpus callosum

- Polymicrogyric appearance and disposition in the left transitional frontal-parietal area

- Hypoplasia of the pituitary stalk, the optic chiasm, and both optic nerves (Figure 1)

Topiramate was started, with excellent seizure control. Endocrine testing revealed low insulin-like growth factor-1 (IGF-1) and growth hormone (GH) levels while the rest of the hypothalamic-pituitary function tests were normal.

**Figure 2:**
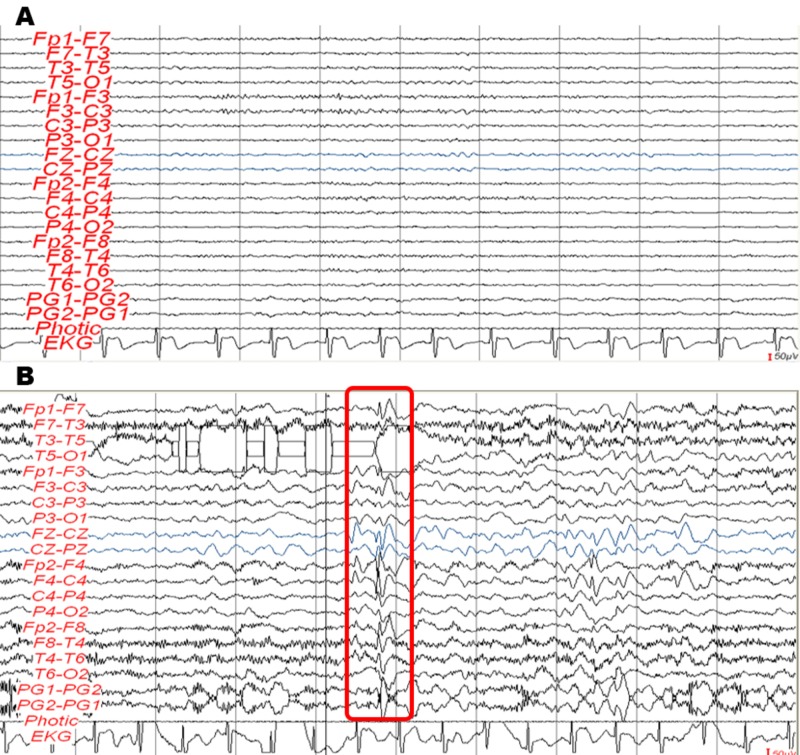
Electroencephalographic findings Panel A: Resting state. Asymmetric and asynchronic basal rhythm. Alpha-range frequency of 8 Hz and left theta range of 6 Hz. Panel B: Hyperventilation stimulation. Increased epileptiform activity. Bifrontal spike-and-wave complexes in the right frontal lobe (red rectangle).

As part of seizure evaluation, an electroencephalogram (EEG) was performed; relevant findings included asymmetry of right hemisphere electrical activity and epileptiform discharges with a right-frontal focus (Figure 2).

Due to the presence of the characteristic midline abnormalities along with cortical dysplasia, a diagnosis of septo-optic dysplasia plus was made.

## Discussion

The term SOD-plus was suggested by Miller in 2000 to differentiate those patients whose global development delay and motor deficits couldn’t be explained by corpus callosum hypoplasia alone and may manifest other underrecognized cortical malformations [2], such as schizencephaly, polymicrogyria, and gray matter heterotopias [9].

A previous case series of 17 patients with SOD found that 6% of the patients met the criteria for classic SOD, 76% of SOD-plus and 18% of SOD-like, with normal septum pellucidum and corpus callosum in the absence of any cortical abnormalities [9]. In our patient, the SOD-plus criteria were met since the three components of the classic triad were described in the presence of schizencephaly and polymicrogyria as the recognized cortical malformations, an association described in 18% of SOD-plus cases [9].

SOD has a wide clinical heterogeneity, from asymptomatic to severe disabling symptoms [1]. The most frequently described clinical manifestations include visual manifestations (85%-90%), intellectual delay (42.5%-60%), epileptic seizures (27.5%-55%), and endocrine disorders (50%-55%) [10-11]. Neurologically, one of the most feared complications is drug-resistant epilepsy, in which focal cortical dysplasia is the most common cause in this population [1]. In this case, imaging confirmed the presence of focal cortical dysplasia, but epileptic seizures were controlled with two drugs.

Most endocrine findings are diagnosed at an early age, with central hypothyroidism (70%) being the most frequent manifestation, followed by GH deficiency (55%), secondary/tertiary adrenal insufficiency (50%), and central diabetes insipidus (30%) [11]. In our case, the most important finding was GH deficiency, which conditioned short stature. However, lifelong monitoring is suggested since more hormonal deficits may appear [12].

## Conclusions

SOD-plus is a syndrome characterized by the original triad described by de Morsier along with other cortical malformations. A high degree of suspicion is required to make the diagnosis and should be included in the differential diagnosis of young adult patients with a history of ophthalmologic, endocrine, and neurologic abnormalities.
